# Effectiveness of Gilteritinib Beyond Second‐Line Therapy in Relapsed/Refractory *FLT3* ‐Mutated Acute Myeloid Leukemia: A Real‐World Multicenter Study of 171 Patients

**DOI:** 10.1002/ajh.70142

**Published:** 2025-11-22

**Authors:** Matteo Molica, Gema Miralles, Richard Dillon, Mario Annunziata, Faisal Basheer, Juan M. Bergua, Ferran Vall‐Llovera Calmet, Victoria Campbell, Denis Cetin, Gaetano Cimino, Giulia Ciotti, Andrea Corbingi, Laura De Fazio, Hasim Atakan Erol, Vincenzo Federico, Cristina Gil, Yasa Gul Mutlu, Amaia Balerdi Malcorra, Sabrina Mariani, Carla Mazzone, Antonino Mulè, Gerardo Musuraca, Anjum Khan, Mariana Norata, Jenny O'Nions, Fanny Erika Palumbo, Cristina Papayannidis, Anna Lina Piccioni, Maria Teresa Olave Rubio, Jackeline Sanchez‐Tovar, Istemi Serin, Alessandra Serrao, Omur Gokmen Sevindik, Giuseppe Sucato, Marina Aurora Urbano, Calogero Vetro, Susana Vives, Marco Rossi, Maria Paola Martelli, Pau Montesinos, Jad Othman, Salvatore Perrone

**Affiliations:** ^1^ Department of Hematology‐Oncology Azienda Universitaria Ospedaliera Renato Dulbecco Catanzaro Italy; ^2^ Hospital General de Elche Alicante Spain; ^3^ Department of Medical and Molecular Genetics King's College London London UK; ^4^ Hematology Unit Cardarelli Hospital Naples Italy; ^5^ Department of Haematology Addenbrooke's Hospital Cambridge UK; ^6^ Department of Hematology Hospital San Pedro de Alcántara Cáceres Spain; ^7^ Hematology Department Hospital Universitari Mútua Terrassa Terrassa Spain; ^8^ Western General Hospital Edinburgh UK; ^9^ Department of Hematology Ege University School of Medicine Izmir Turkey; ^10^ Hematology, CREO, Department of Medicine and Surgery University of Perugia and Azienda Ospedaliera di Perugia, Perugia, Italy Perugia Italy; ^11^ Department of Oncology UOC Oncohematology, Istituto Oncologico Veneto (IOV) IRCCS Padova Italy; ^12^ Department of Hematology S. M. Goretti Hospital, Polo Universitario Pontino Latina Italy; ^13^ Department of Hematology Kocaeli University Faculty of Medicine Kocaeli Turkey; ^14^ Hematology and Transplant Unit Vito Fazzi Hospital Lecce Italy; ^15^ Hospital General de Alicante Alicante Spain; ^16^ Department of Hematology Gaziantep City Hospital Gaziantep Turkey; ^17^ Hospital Universitario Cruces San Vicente de Barakaldo Bizkaia Spain; ^18^ Department of Hematology University Hospital Sant'Andrea, Sapienza University of Rome Rome Italy; ^19^ Hematology Unit Sant’Eugenio Hospital Rome Italy; ^20^ Hematology Unit A.O.O.R. Villa Sofia—Cervello Palermo Italy; ^21^ IRST “Dino Amadori” IRCCS Meldola Italy; ^22^ Department of Haematology Leeds Teaching Hospitals Trust Leeds UK; ^23^ University College London Hospital NHS Foundation Trust London UK; ^24^ Division of Hematology AOU Policlinico G. Rodolico‐San Marco Catania Italy; ^25^ IRCCS Azienda Ospedaliero‐Universitaria di Bologna, Istituto di Ematologia “Seràgnoli” Bologna Italy; ^26^ Department of Hematology San Giovanni‐Addolorata Hospital Rome Italy; ^27^ Hospital Clinico Universitario Lorenzo Blesa Zaragoza Spain; ^28^ Hospital Rio Carrión, Complejo Asistencial de Palencia Palencia Spain; ^29^ Department of Hematology Istanbul Basaksehir Cam and Sakura City Hospital Istanbul Turkey; ^30^ Hematology Unit San Camillo Hospital Rome Italy; ^31^ Clinic of Hematology Istanbul Florence Nightingale Hospital Istanbul Turkey; ^32^ Hematology Unit University Hospital Paolo Giaccone Palermo Italy; ^33^ Hematology Unit A. Perrino Hospital Brindisi Italy; ^34^ Hematology and Bone Marrow Transplantation Unit (BMTU), Hospital of Bolzano (SABES‐ASDAA) Teaching Hospital of Paracelsus Medical University (PMU) Bolzano Italy; ^35^ Hospital U. Germans Trias i Pujol ICO Badalona Spain; ^36^ Hematology Department La Fe University and Polytechnic Hospital Valencia Spain

**Keywords:** AML, *FLT3* mutation, gilteritinib, hematopoietic stem cell transplantation, real‐world evidence, third‐line therapy

## Abstract

Gilteritinib is a selective FLT3 inhibitor approved for the treatment of relapsed or refractory (R/R) *FLT3*‐mutated acute myeloid leukemia (AML) following ≥ 1 prior line of therapy. However, data on its effectiveness in later‐line settings is limited. We conducted a multicenter, retrospective study including 171 adult patients with R/R *FLT3*‐mutated AML who received gilteritinib as third‐line or beyond between August 2017 and March 2024 across centers in Italy, Spain, the United Kingdom, and Turkey. The primary endpoint was overall survival (OS). Secondary endpoints included overall response rate (ORR), composite complete remission (cCR), duration of response. Among the 171 patients, 84% carried *FLT3*‐ITD mutations and 26% had received ≥ 3 prior lines of therapy. The cCR rate was 28%, and ORR was 47%. Patients who were younger and presented with relapsed (vs. refractory) disease had better outcomes. Prior exposure to venetoclax or allogeneic hematopoietic stem cell transplantation (HSCT) was associated with inferior response. Gilteritinib enabled HSCT in 12% of patients. Median OS was 7.1 months (95% CI, 5.9–10.1), and in Cox‐regression analysis was significantly improved among responders and those who underwent HSCT (median OS: 21.5 months; 95% CI, 12.8–NR). Prior venetoclax exposure was associated with shorter survival (5.7 months; 95% CI, 5.1–8.8). On multivariate analysis, previous exposure to venetoclax and FLT3 inhibitors was the strongest predictor of reduced response rates. Despite heavy pretreatment, gilteritinib retained clinically relevant activity in later‐line R/R *FLT3*‐mutated AML. Its use beyond second‐line may serve as a bridge to HSCT in selected patients. Resistance mechanisms, particularly following venetoclax, remain a therapeutic challenge. These data support the continued use of gilteritinib beyond second‐line and highlight the need for prospective studies to optimize sequencing strategies.

## Graphical abstract

This multicentric retrospective study evaluated the efficacy of gilteritinib in a cohort of 171 adult patients with relapsed/refractory (R/R) acute myeloid leukemia (AML) harboring a FLT3 mutation, who had previously received at least two lines of therapy. Notably, 26% of the patients had received three or more prior lines of treatment. The analysis of the entire cohort showed a median overall survival (OS) of 7.1 months. The two most important prognostic factors for improved survival were: Quality of Response to Gilteritinib: Patients who achieved a complete response (CR/CRi/MLFS) had a significantly longer median OS of 16.3 months, compared to 6.7 months for those who achieved a partial response (PR), and 4.8 months for patients with stable or nonresponsive disease (SD/NR) (*p* < 0.001). Allogeneic stem cell transplant (HSCT): Patients who underwent an allogeneic transplant after gilteritinib treatment showed a median OS of 16.3 months, a value markedly superior to the 6.5 months recorded in the nontransplanted patient group (*p* < 0.001). In conclusion, achieving a deep response to gilteritinib and the feasibility of proceeding to an allogeneic transplant are the main predictors of a favorable outcome in this heavily pretreated patient population. Gilteritinib is affective also in the third or further lines of treatment.

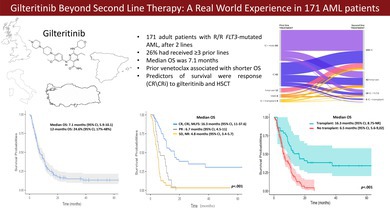

## 1 Introduction

Acute myeloid leukemia (AML) is a biologically and clinically heterogeneous hematologic malignancy characterized by clonal expansion of myeloid progenitors and impaired hematopoietic differentiation. The disease predominantly affects older adults and is associated with a poor prognosis, especially in the presence of adverse‐risk cytogenetic or molecular features. In Western countries, the age‐adjusted incidence of AML is approximately 4.3 per 100 000 persons annually [1].

Among the most clinically relevant molecular aberrations in AML, internal tandem duplications in the FMS‐like tyrosine kinase 3 gene (*FLT3*‐ITD) are found in approximately 30% of cytogenetically normal AML cases [2]. These mutations are often associated with hyperleukocytosis [3] and confer an increased risk of relapse and reduced overall survival (OS) [4].

The therapeutic landscape of *FLT3*‐mutated AML has evolved considerably with the development of FLT3‐targeted agents [5]. In the frontline setting, the addition of midostaurin [6] or quizartinib [7] to induction chemotherapy has improved survival, and their integration into standard care is reflected in recent guidelines. The 2022 European LeukemiaNet (ELN) recommendations reclassified *FLT3*‐ITD‐positive AML, regardless of *NPM1* status, as intermediate risk in the absence of other adverse features [8]. This update differs from the 2017 ELN classification, where *FLT3*‐ITD^high/*NPM1*^wt was considered adverse risk [9], underlining the diminishing prognostic role of allelic ratio [10, 11].

In the relapsed/refractory (R/R) setting, the second‐generation FLT3 inhibitor gilteritinib demonstrated superior efficacy over salvage chemotherapy in the Phase 3 ADMIRAL trial, significantly improving both response rates and OS in *FLT3*‐mutated AML [12]. Gilteritinib is now the standard of care following relapse, yet real‐world clinical trajectories often diverge from trial settings [13, 14]. Disease evolution may lead to late acquisition of *FLT3* mutations, sometimes at second relapse or following prior treatment with venetoclax or other targeted therapies [15, 16].

Despite regulatory approval for use after ≥ 1 prior line, the use of gilteritinib beyond second‐line remains underexplored in real‐life scenarios. A subset of patients may receive this agent in later lines due to delayed *FLT3* mutation detection, clonal evolution, or limited treatment options. In this context, we conducted a multinational, retrospective analysis to evaluate the real‐world outcomes of gilteritinib administered as third‐line or beyond therapy in patients with R/R *FLT3*‐mutated AML. Our aim was to inform clinical practice and support rational sequencing strategies in this high‐risk population.

## 2 Methods

### 2.1 Study Design and Setting

We conducted a multinational, multicenter, retrospective cohort study across academic institutions in Italy, Spain (from the PETHEMA cooperative group), the United Kingdom (extracted from a previously published cohort), and Turkey. Adult patients with relapsed or refractory (R/R) *FLT3*‐mutated AML treated with gilteritinib as third‐line or later therapy between August 2017 and December 2024 were included.

### 2.2 Eligibility Criteria and Data Collection

Patients were eligible if they had a confirmed diagnosis of AML according to WHO 2016 criteria, harbored a *FLT3* mutation—either ITD or tyrosine kinase domain (TKD)—and had received at least two prior lines of AML‐directed therapy. Gilteritinib was administered in accordance with the approved label and institutional practice.

Demographic, clinical, molecular, and treatment‐related data were retrospectively extracted from electronic medical records. Collected variables included age, sex, AML status (relapsed vs. refractory), *FLT3* mutation subtype, prior treatments (including venetoclax, FLT3 inhibitors, and hematopoietic stem cell transplantation [HSCT]), treatment response, and survival outcomes.

### 2.3 Study Endpoints and Definitions

The primary endpoint was OS, defined as the time from initiation of gilteritinib to death from any cause. Secondary endpoints included: event‐free survival (EFS) defined as the time from treatment start to primary refractory disease, relapse after response, or death; overall response rate (ORR) defined as the proportion of patients achieving complete remission (CR), CR with incomplete hematologic recovery (CRi), morphological leukemia‐free state (MLFS), or partial response (PR); composite CR (cCR) defined as the sum of CR and CRi; and duration of response (DoR) defined as the time from first documented response to relapse or death.

Response assessments were performed according to the 2017 ELN criteria. The acquisition of *FLT3* mutations at relapse, and prior exposure to targeted agents, were recorded as additional covariates.

### 2.4 Statistical Analysis

Categorical variables were compared using Fisher's exact test. Continuous variables were analyzed using Student's *t*‐test or the Mann–Whitney *U* test, as appropriate. Dichotomous predictors were evaluated using univariate logistic regression.

Survival analyses were conducted using the Kaplan–Meier method. Differences between survival curves were assessed using the log‐rank test. Cox proportional hazards models were employed for multivariate analysis to identify independent predictors of OS and EFS. Covariates included: age group (< 50, 50–65, > 65 years), sex, relapsed versus refractory disease, *FLT3* mutation subtype (ITD vs. TKD), number of prior therapy lines (2 vs. > 2), prior exposure to FLT3 inhibitors or venetoclax or HSCT, emergence of new *FLT3* mutations, best response to gilteritinib and receipt of HSCT post‐gilteritinib. All clinically relevant variables were considered for multivariable analysis, regardless of their statistical significance in univariate testing. This approach was chosen to allow simultaneous adjustment for potential confounders and to account for the biological plausibility of each covariate. HSCT was considered a time‐dependent variable and patients receiving HSCT or not, were stratified according to Simon–Makuch analysis (in R).

Hazard ratios (HRs) and 95% confidence intervals (CIs) were reported. Forest plots were generated using Python 3.9 (Google Colab environment).

### 2.5 Ethical Considerations

The study was conducted in accordance with the Declaration of Helsinki (1975, as revised in 2008) and was approved by the institutional review board of the coordinating center (Protocol ID: GILT‐01, No. 2529). Written informed consent was obtained from all participants, or the requirement for consent was waived, in line with local ethical guidelines.

## 3 Results

### 3.1 Patient Characteristics

A total of 171 patients were included across participating centers in Italy, Spain (PETHEMA group), the United Kingdom, and Turkey. All patients received gilteritinib single agent, as third‐line or later therapy between August 2017 and December 2024. The median age at treatment initiation was 54.9 years (range, 22.7–77.6), with 30% aged > 65 years.

**FIGURE 1:**
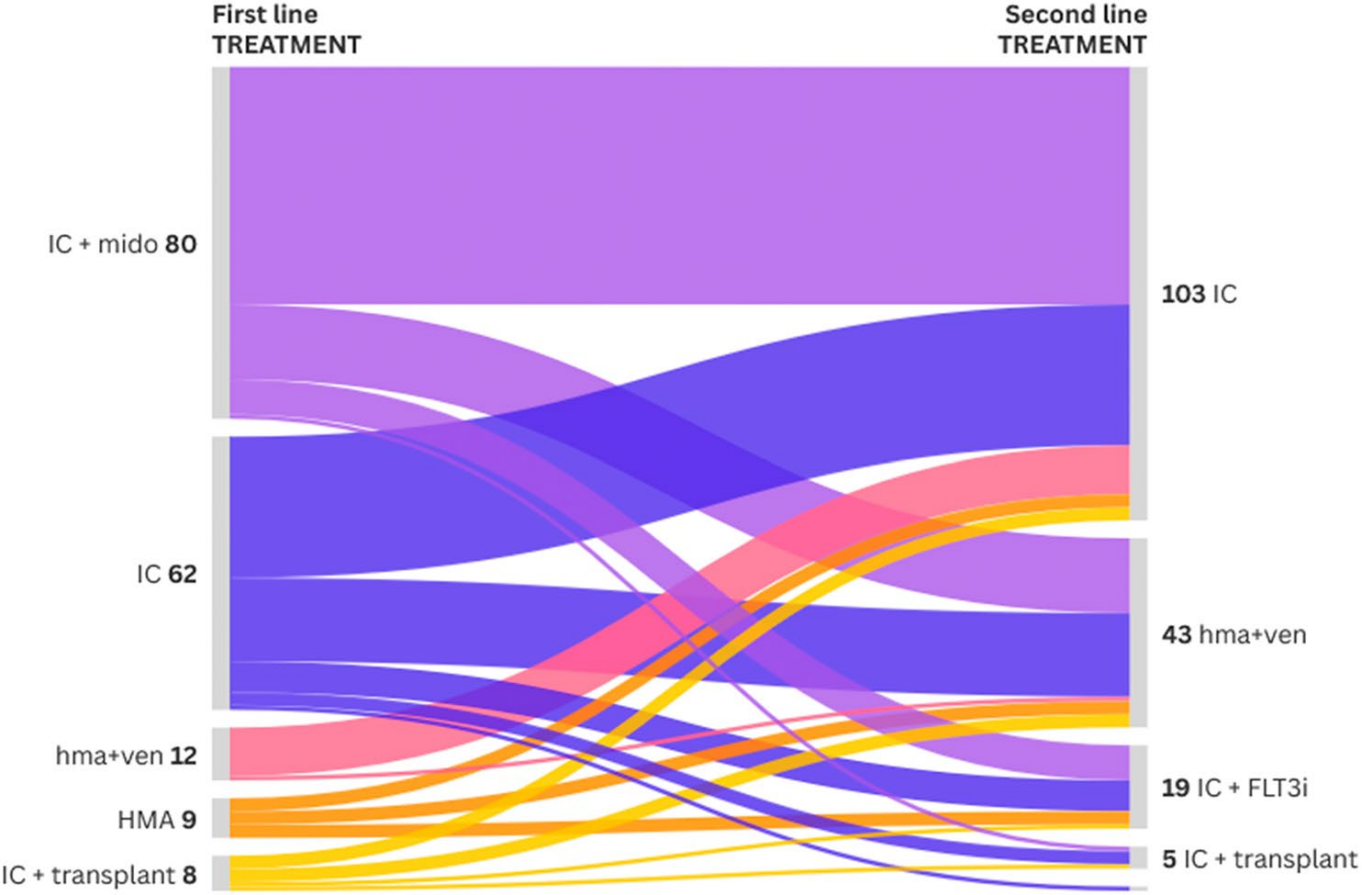
Sankey graphic showing the first (on the left side) and second line (on the right side) of treatment received before gilteritinib (made with Flourish). One patient indicated as HMA + ven in first and second line, indeed received an experimental Bcl2/Bclx inhibitor. [Color figure can be viewed at wileyonlinelibrary.com]

*FLT3*‐ITD mutations were detected in 144 patients (84%), while 115 (67%) had *FLT3* mutations documented at diagnosis. Overall, 110 patients (64%) had relapsed AML, and 61 (36%) presented with refractory disease. Regarding first‐line therapy, 36% of patients received intensive chemotherapy (IC), 48% received IC plus midostaurin, 7% received hypomethylating agents (HMAs) plus venetoclax, 5% were treated with HMAs alone, and 5% underwent upfront allogeneic HSCT. For second‐line treatment, 61% received IC, 11% IC plus a FLT3 inhibitor, 25% HMA plus venetoclax, and 3% underwent allogeneic HSCT (Figures 1 and S6).

Prior exposure to FLT3 inhibitors was reported in 98 patients (57%), and 59 patients (35%) had received venetoclax. Forty‐two patients (26%) received ≥ 3 prior lines of therapy; this subgroup was slightly younger (median age 50.3 vs. 61.2 years) and more frequently exposed to multiple FLT3 inhibitors (10%) or gemtuzumab ozogamicin (24%) (Table 1).

**TABLE 1:** Baseline characteristics of AML patients at the beginning of gilteritinib treatment.

Patients characteristic	*N* = 171
Median age (in years)	54.9 (22.7–77.6)
Age groups (in years)	
< 50	56 (33%)
≥ 50 ≤ 65	64 (37%)
> 65	51 (30%)
Sex	
Male	87 (51%)
Female	84 (49%)
*FLT3* mutation at any time	
ITD	144 (84%)
TKD	20 (12%)
ITD + TKD	7 (4.1%)
Onset of *FLT3* mutation	
New onset	56 (33%)
At diagnosis	115 (67%)
Disease status	
Relapse	110 (64%)
Refractory	61 (36%)
First line treatment	
Intensive chemotherapy (IC) + midostaurin	80 (48%)
IC	62 (36%)
IC + transplant	8 (5%)
Hypomethylating agents (HMA)‐venetoclax	12 (7%)
HMA	9 (5%)
Second line treatment	
IC	103 (61%)
IC + FLT3 inhibitor	19 (11%)
IC + transplant	5 (3%)
Hypomethylating agents (HMA)‐venetoclax	43 (25%)
Lines of therapy before gilteritinib	
2	129 (74%)
≥ 3	42 (26%)
FLT3 inhibitor before gilteritinib	
Yes	98 (57%)
No	73 (43%)
HMA + venetoclax before gilteritinib	
Yes	59 (35%)
No	112 (65%)

### 3.2 Treatment Response

Among 162 evaluable patients, CR was achieved in 40 (24.7%), CRi in 8 (5%), resulting in a cCR rate of 28%. Additional responses included MLFS in 2% and PR in 15%, yielding an ORR of 47% (Table 2).

**TABLE 2:** Rate of remission and outcome.

Characteristic	All patients, *N* = 171
Median number cycles gilteritinib (range)	5 (1–22)
Response	47%
ORR	
CR	40 (24%)
CRi	8 (5%)
MLFS	3 (2%)
PR	26 (16%)
Refractory disease	85 (52%)
Death before response assessment	9 (5%)
Allogeneic transplant	21 (12%)
Survival	
Day‐30 mortality	2%
Day‐60 mortality	13%
12‐month survival	24.6% (95% CI, 17–32)
Median survival (month)	7.1 (95% CI, 5.9–10.1)

Nine patients (5%) died before response assessment, predominantly due to infection (*n* = 6) or severe hemorrhage (*n* = 3). The median number of gilteritinib cycles administered was 5 (range, 1–22), with a median treatment duration of 3.04 months (95% CI, 0.19–20.8). Reasons for treatment discontinuation included refractory disease (36%), death (15%), toxicity (10%), and transition to HSCT (12%). When considering the whole population of 171 patients, CR was achieved by 23%, CRi by 4.7%, resulting in a cCR rate of 27.7%.

**FIGURE 2:**
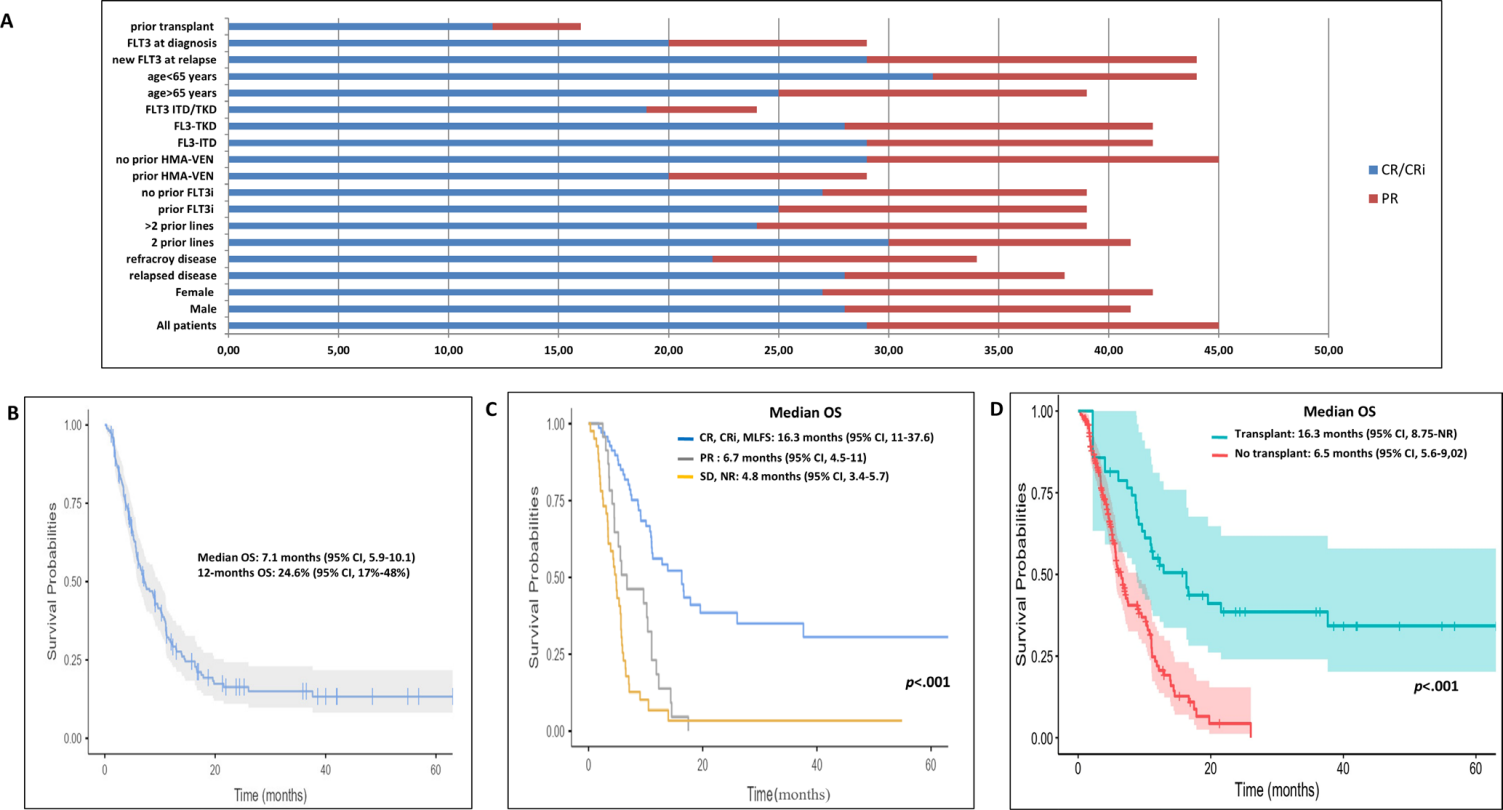
(A) Best response achieved by clinical and genomic subgroups, on the *x*‐axis is shown the % of patients. (B) Kaplan–Meier plot of OS, entire cohort of patients. (C) Kaplan–Meier plot of OS for patients treated with gilteritinib, stratified per best response achieved. (D) Simon–Makuch plots of OS for patients treated with gilteritinib, stratified per receiving (or not) allogeneic transplant. [Color figure can be viewed at wileyonlinelibrary.com]

Among patients achieving cCR, the median time to best response was 1.52 months (95% CI, 0.59–6.16), with 77% receiving ≥ 4 cycles. Median duration of response was 9.62 months (95% CI, 1.04–26.03) for CR/CRi and 5.75 months (95% CI, 0.94–12.17) for PR. Response rates were higher in patients aged < 65 versus ≥ 65 years (32% vs. 25%; *p =* 0.17), in relapsed versus refractory disease (28% vs. 22%, *p* = 0.69), and in those treated in third versus later lines (30% vs. 24%; *p* = 0.23). Prior exposure to FLT3 inhibitors did not impact cCR rates (27% vs. 25%, *p =* 0.73), whereas previous treatment with HMA plus venetoclax was associated with reduced response (20% vs. 29%; *p =* 0.43). Notably, patients with *FLT3* mutations detected at relapse achieved higher cCR rates than those with baseline *FLT3*‐mutant disease (29% vs. 20%; *p =* 0.07). Among previously transplanted patients, cCR was observed in only 12% (Figure 2A).

On multivariate logistic regression, prior exposure to venetoclax (*p* = 0.023; 95% CI, 0.81–5.95) and prior use of FLT3 inhibitors (*p* = 0.041; 95% CI, 1.04–6.61) were independently associated with lower response probability (Figure S1).

Twenty‐one patients (12%) underwent allogeneic HSCT following gilteritinib. Most were < 65 years (71%), had relapsed disease (67%), *FLT3*‐ITD mutations (81%), and had not received HMA‐venetoclax combinations (76%). The median time from gilteritinib initiation to HSCT was 3.86 months (95% CI, 1.09–15.08). Among patients achieving CR/CRi, the 12‐month cumulative incidence of relapse was 39%, compared to 76% among those achieving MLFS or PR.

### 3.3 Survival Outcomes

At a median follow‐up of 18.6 months, Day‐30 and Day‐60 mortality rates were 5% and 9%, respectively. The median OS for the entire cohort was 7.1 months (95% CI, 5.9–10.1), with 12‐ and 18‐month OS rates of 25% and 12%, respectively (Figure 2B).

**FIGURE 3:**
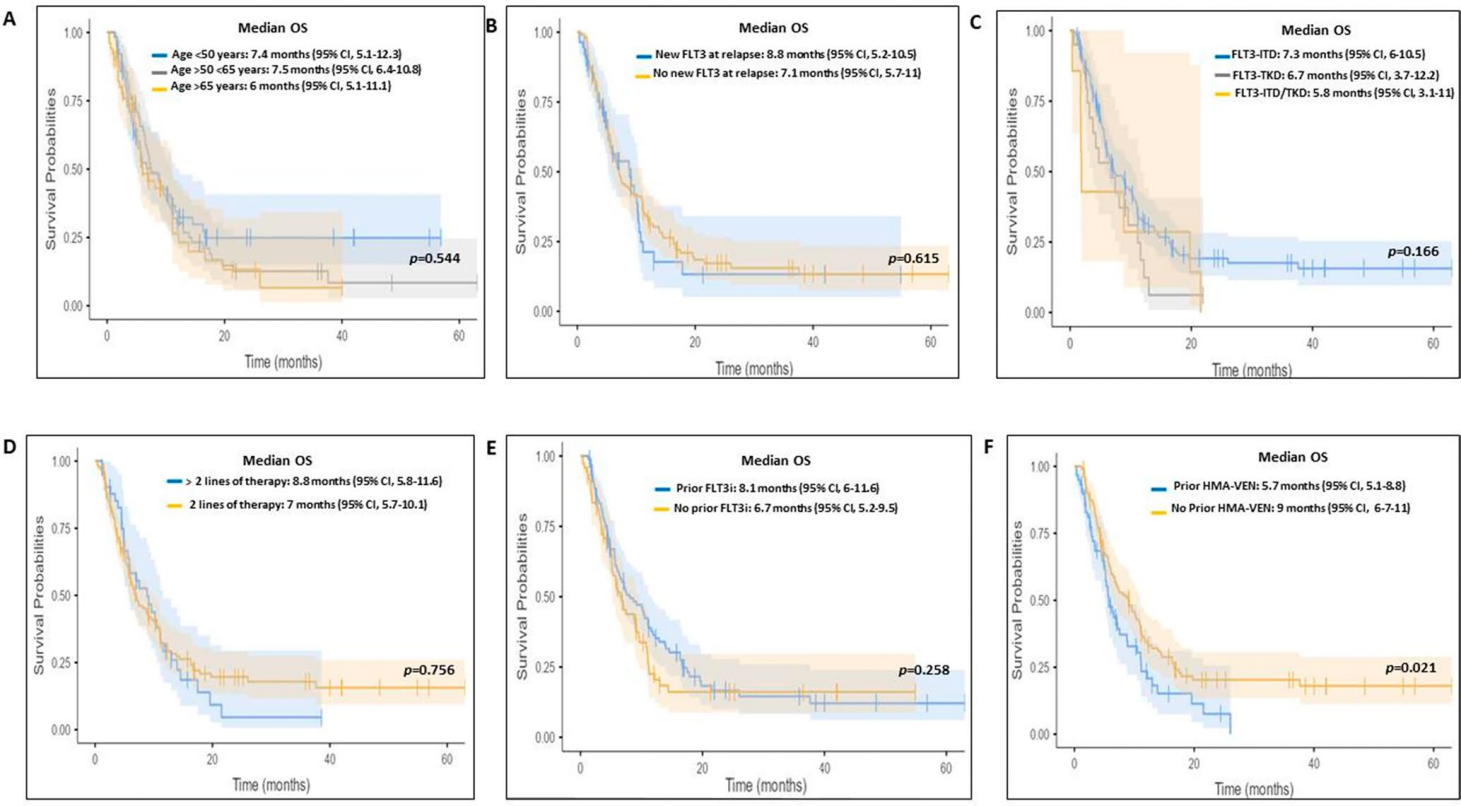
Kaplan–Meier curves of OS were stratified for: (A) Age sub‐groups, (B) new onset FLT3mutation, (C) type of FLT3 mutation, (D) number of previous lines of therapy, (E) previous exposure to FLT3 inhibitors, (F) previous exposure to HMA + venetoclax. [Color figure can be viewed at wileyonlinelibrary.com]

Responders to gilteritinib had significantly improved survival compared to nonresponders. The 12‐month OS was 59% in patients achieving CR and 54% in those with CRi (Figure 2C). Patients bridged to HSCT had a markedly superior median OS of 16.3 months (95% CI, 8.75–NR) versus 6.5 months (95% CI, 5.6–9.02), for those not undergoing HSCT (*p* < 0.001). In univariate analysis, age, sex, *FLT3* mutation subtype (ITD vs. TKD), number of prior therapies, prior exposure to FLT3 inhibitors, or acquisition of *FLT3* mutation at relapse were not significantly associated with OS. However, prior venetoclax exposure correlated with inferior survival (median OS 5.7 vs. 9.0 months; *p* = 0.021) (Figure 3).

In multivariate Cox regression, we were unable to identify risk factors at the start of gilteritinib (Figure S2A); the only independent predictors of improved OS were achievement of CR/CRi and post‐gilteritinib HSCT (Figure S2B). Median EFS was 3.1 months (95% CI, 2.8–3.7) (Figure S3). Neither prior FLT3 inhibitor nor venetoclax exposure significantly impacted EFS in univariate (Figure S4 and Table 1) or multivariate analysis (Figure S5).

## 4 Discussion

To our knowledge, this is the largest real‐world study specifically evaluating the efficacy of gilteritinib beyond the second‐line setting in patients with R/R *FLT3*‐mutated AML. Gilteritinib has been established as the standard of care for R/R *FLT3*‐mutated AML following the results of the Phase 3 ADMIRAL trial, which demonstrated superior OS and response rates compared to salvage chemotherapy [12]. However, data supporting its use beyond the second line remain limited, and current treatment guidelines are largely based on second‐line evidence [17, 18],

In clinical practice, delayed detection of *FLT3* mutations, clonal evolution, or lack of earlier access to targeted agents often results in gilteritinib being used in more advanced settings. In our multinational cohort of 171 patients, over a quarter received gilteritinib in the fourth line or beyond, with a substantial proportion pretreated with venetoclax (35%) and prior FLT3 inhibitors (57%).

Despite the heavily pretreated nature of this population, gilteritinib demonstrated clinically meaningful activity. The cCR rate was 28%, with an ORR of 47%. These features compare favorably with the ADMIRAL trial, where the CR + CRh rate was 34% [12]. While we observed a similar CR rate (23% vs. 21.1% in ADMIRAL), the median OS of 7.1 months in our cohort aligns with other real‐world studies, including those from ALFA‐FILO (6.4 months), Israel (8.0 months), the United Kingdom (9.5 months), Spain (4.7 months), and Italy (7.1 months) [19, 20, 21, 22, 23].

Our data support the hypothesis that gilteritinib retains anti‐leukemic activity even in later lines of therapy. However, outcomes are clearly influenced by disease biology and prior treatments. Notably, prior exposure to venetoclax was associated with reduced response and shorter survival, consistent with emerging evidence suggesting cross‐resistance mechanisms and limited salvage efficacy post‐venetoclax [21, 24, 25]. Similarly, patients previously undergoing HSCT showed inferior responses, possibly reflecting more aggressive or chemo‐resistant disease.

Multivariate analysis identified HSCT following gilteritinib as the only independent predictor of prolonged OS. Patients who underwent HSCT had a median OS of 21.5 months, underscoring the value of gilteritinib as a bridge‐to‐transplant strategy even in advanced settings. Nevertheless, only 12% of patients were ultimately transplanted, likely due to rapid progression, comorbidities, or limited donor availability.

Interestingly, neither prior FLT3 inhibitor exposure nor the number of prior therapy lines significantly influenced response rates or survival in multivariate models. This may suggest that gilteritinib remains active despite prior *FLT3*‐targeted therapy, though the lack of prospective molecular data limits definitive conclusions [26, 27, 28]. In our cohort, 62 patients received intensive chemotherapy and 8 patients received IC and HSCT in the frontline setting. Moreover, 66 patients were diagnosed before 2019 when midostaurin was not easily available in Europe, so it could explain its low use. For *FLT3*‐inhibitors exposure, most patients received midostaurin and only three patients received quizartinib.

The absence of measurable residual disease (MRD) assessment is a notable limitation of our study. Sensitive techniques for *FLT3*‐ITD monitoring, such as PCR‐based or NGS assays, remain investigational and were not routinely available across centers [29, 30, 31]. Additionally, retrospective study design and treatment heterogeneity are inherent limitations, though mitigated by the multicenter nature and sample size.

Ongoing trials are evaluating triplet regimens combining FLT3 inhibitors, venetoclax, and HMAs in both newly diagnosed and R/R AML [32, 33, 34, 35]. Such strategies may eventually redefine the therapeutic landscape, but for now, our findings support the continued use of gilteritinib beyond the second‐line setting, particularly as a bridge to transplantation in selected patients.

## 5 Conclusions

In conclusion, gilteritinib demonstrates sustained clinical activity in heavily pretreated *FLT3*‐mutated AML patients and may offer a bridge to curative therapies even when used beyond second line. These real‐world data underscore the importance of individualized treatment sequencing and support future prospective studies to optimize the integration of FLT3 inhibitors in salvage settings.

## Author Contributions

S.P. and M.M. conceptualized the study and coordinated data collection. All authors collected data, reviewed, edited, and approved the final version of the manuscript.

## Conflicts of Interest

A.K. honoraria/consultancy: AbbVie, Astellas, Incyte, Immedica, Jazz, Medac. Novartis, Pfizer, Otsuka, Servier, Synairgen & TC BioPharm. M.P.M. declares honoraria/consultancy at the scientific advisory board for AbbVie, Amgen, Be‐One, BMS, Delbert, Janssen, Gilead, Novartis, Pfizer, and Jazz Pharmaceuticals. J.O. declares honoraria from Astellas, Jazz and Pfizer. C.V. Honoraria: Jazz; Astellas; Advisory Board: Jazz; AbbVie; BMS; Astellas; Incyte; Amgen. The other authors declare no conflicts of interest.

## Supporting information


**Data S1:** ajh70142‐sup‐0001‐Supinfo1.pdf.

## Data Availability

The data that support the findings of this study are available from the corresponding author upon reasonable request. Separate requests to each study group may be required. Individual participant data have been de‐identified in compliance with applicable privacy regulations.
