# Supplementary material for: Effectiveness of Gilteritinib Beyond Second‐Line Therapy in Relapsed/Refractory FLT3 ‐Mutated Acute Myeloid Leukemia: A Real‐World Multicenter Study of 171 Patients
Source: Am J Hematol. 2025 Nov 22;101(1):89–96. doi: 10.1002/ajh.70142 (PMC12669949; doi:10.1002/ajh.70142)

Effectiveness of gilteritinib beyond second-line therapy in relapsed/refractory *FLT3*-mutated acute myeloid leukemia: A Real-world multicenter study of 171 patients.

Supplemental data

Figure 1 Supplemental. Logistic regression analysis of risk factors associated to the response to gilteritinib.

Logistic regression CR/CRi/MLFS vs. resistant to gilteritinib

Variable	Odds Ratio	IC 95% (lower)	IC 95% (higher)	p
**Intercept**	0.32	0.07	1.42	0.135
**Age 50–65 vs <50 yrs.**	0.81	0.30	2.19	0.671
**Age >65 vs <50 yrs.**	0.80	0.26	2.47	0.698
**Gender Female vs Male**	0.77	0.33	1.82	0.550
**>2 lines before gilteritinib**	0.72	0.25	2.04	0.536
**FLT3-i before gilteritinib (no vs. yes)**	2.62	1.04	6.61	**0.041**
**Venetoclax before (no vs. yes)**	2.19	0.81	5.95	**0.023**
**FLT3 mutation de novo (no vs. yes)**	0.91	0.35	2.36	0.844

Figure 2 Supplemental. Forest plot of the Hazard ratio of risk factors, multivariate analysis (Cox regression) for OS. **A** Risk-factors present at starting gilteritinib. **B** Risk-factors considered during the timeset of the study.

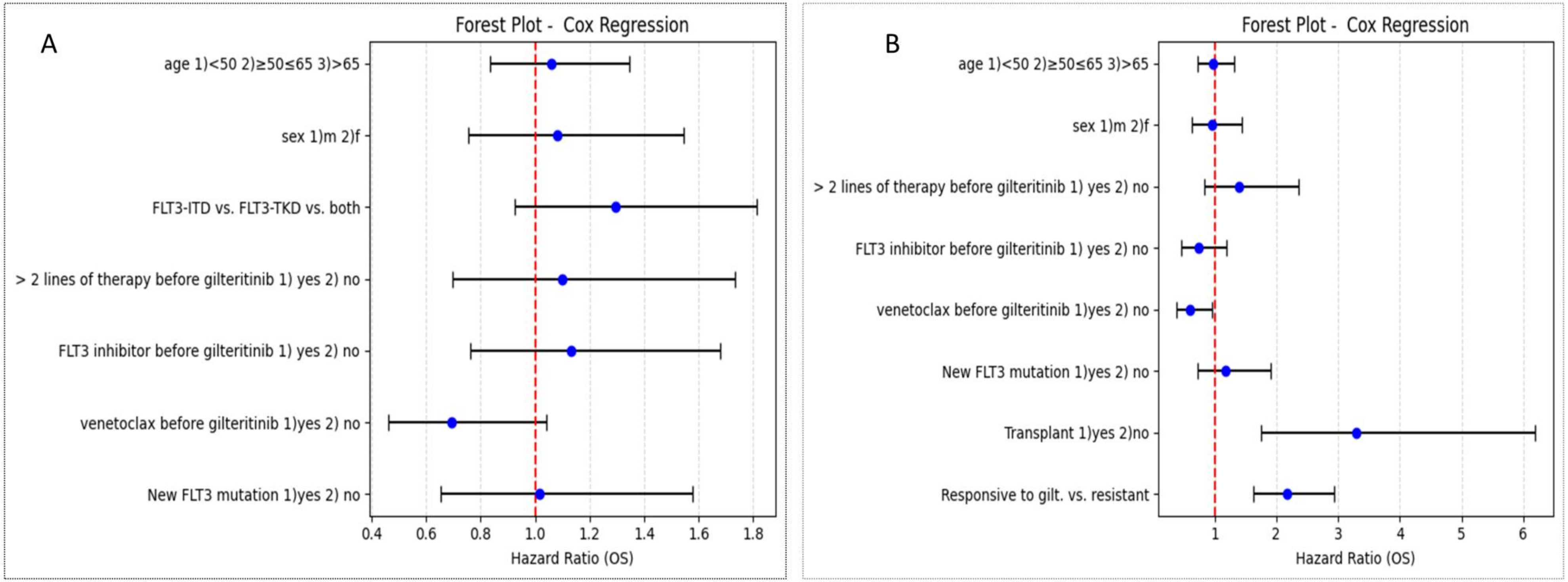

Figure 3 Supplemental. Kaplan Meyers curves of EFS. X-axis: Months.

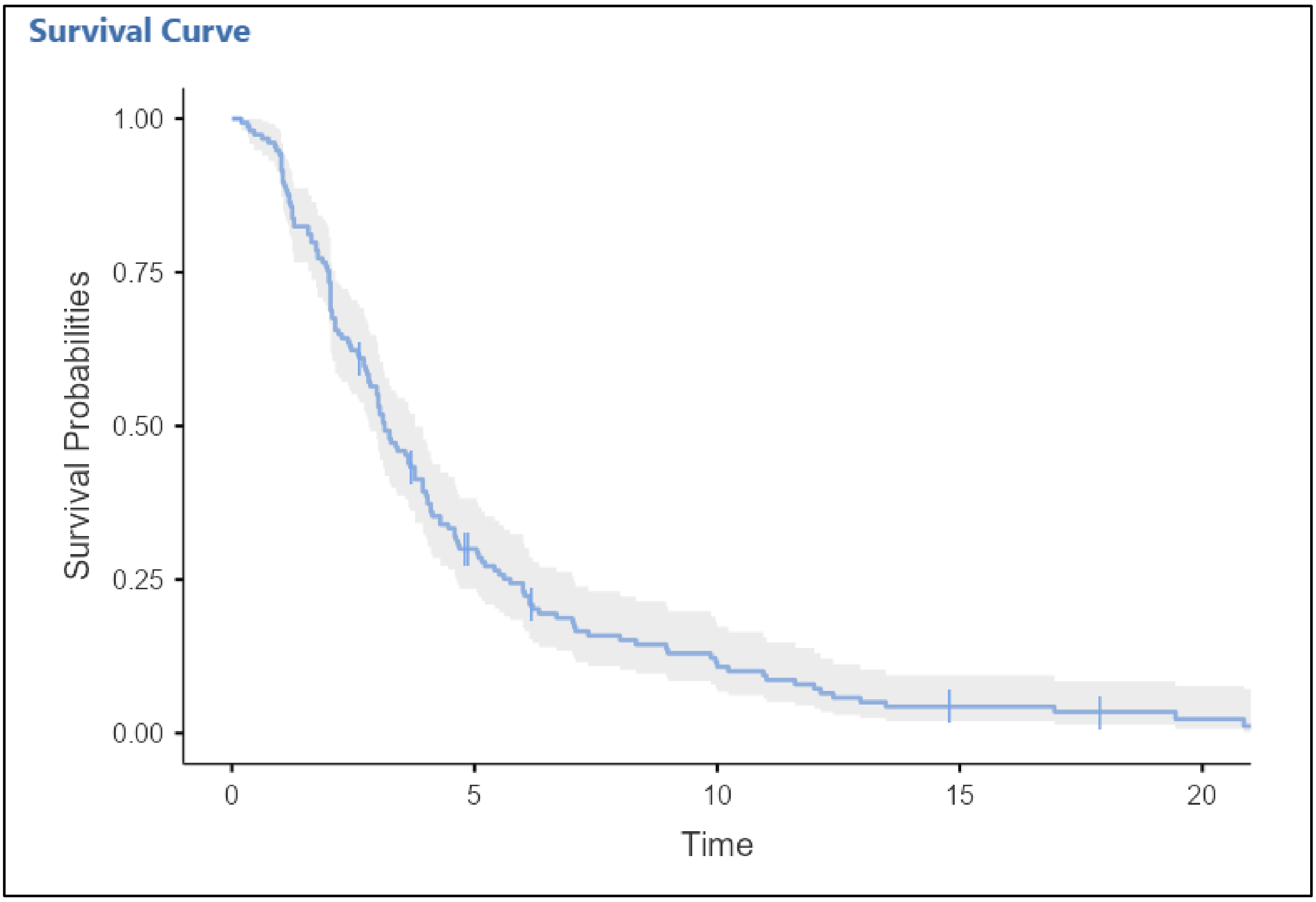

Figure 4 Supplemental. Kaplan Meyers curves of EFS were stratified for: **A** age sub-groups, **B** new onset FLT3mutation, **C** type of FLT3 mutation, **D** number of previous lines of therapy, **E** previous exposure to FLT3-inhibitors, **F** previous exposure to HMA+ venetoclax.

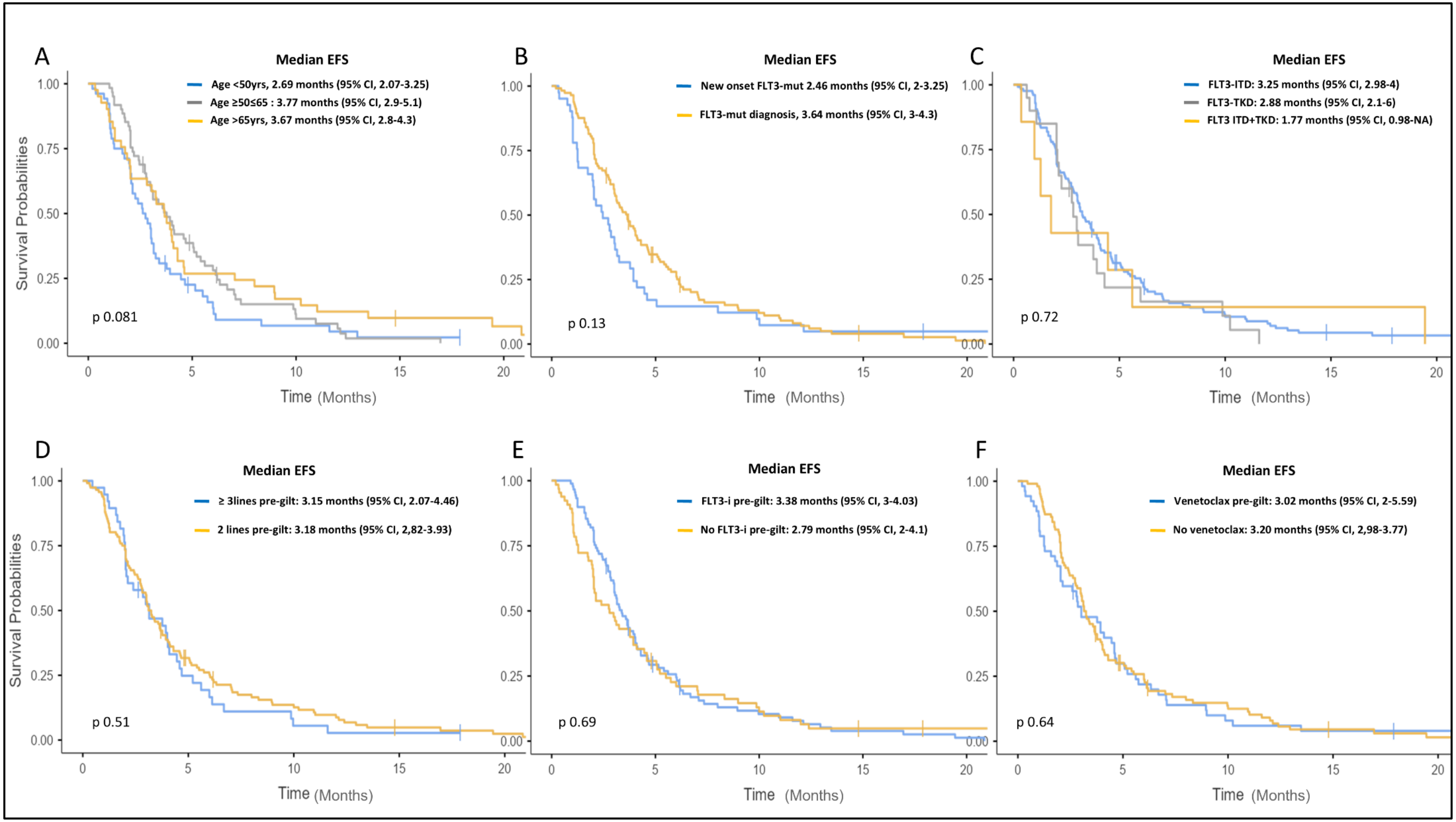

Table 1 Supplemental. Univariate analysis for EFS

	EFS (months)	95% CI	P value
**Entire cohort**	3.1	2.8-3.7	
**Sex**<br>**Female**<br>**Male**	3.15<br>3.25	2.8-4.1<br>2.4-3.9	**0.614**
**Type of *FLT3***<br>**ITD**<br>**TKD**	3.25<br>2.82	2.98-4<br>2.13-6	**0.718**
**Age**<br>**<50 years**<br>**>50<65 years**<br>**>65 years**	2.69<br>3.77<br>3.67	2.97-3.25<br>2.98-5.08<br>2.82-4.30	**0.081**
**Prior FLT3 inhibitor**<br>**Yes**<br>**No**	3.38<br>2.79	3.02-4.03<br>2.03-4.10	**0.692**
**Prior venetoclax**<br>**Yes**<br>**No**	3.02<br>3.2	2.03-4.09<br>2.98-3.77	**0.638**
**Lines of therapy**<br>**2**<br>**>2**	3.49<br>2.95	3.13-4.15<br>2.05-3.89	**0.567**
***FLT3* occurrence**<br>**Baseline**<br>**Relapse**	2.46<br>3.64	2-3.25<br>3.92-4.3	**0.139**
**AML disease**<br>**Relapse**<br>**Refractory**	2.68<br>3.16	2.05-3.41<br>2.95-3.72	**0.567**
**Response**<br>**CR+CRi+MLFS**<br>**Resistant**	4.59<br>2.13	3.28-6<br>1.74-3.25	**<0.001**
**Transplant**<br>**Yes**<br>**No**	20.1<br>3.03	11.8-NR<br>2.71-3.65	**<0.001**

Figure 5 Supplemental Forest plot of the Hazard ratio of risk factors, multivariate analysis (Cox regression) for EFS.

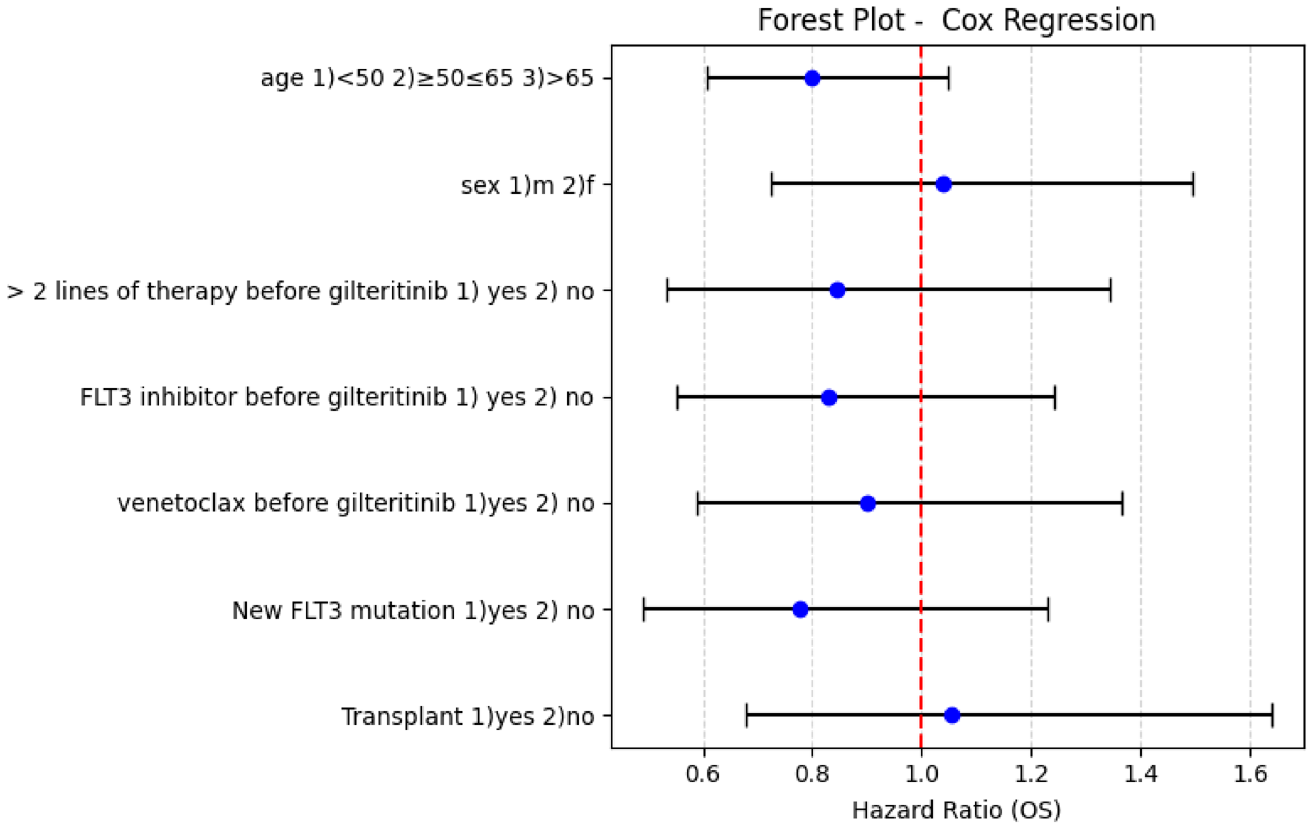

Figure 6 Supplemental Sankey graphic showing the first, second line, and third line of treatment received before gilteritinib for the subgroup of patients that received ≥3 prior lines of therapy before gilteritinib (Made with Flourish).

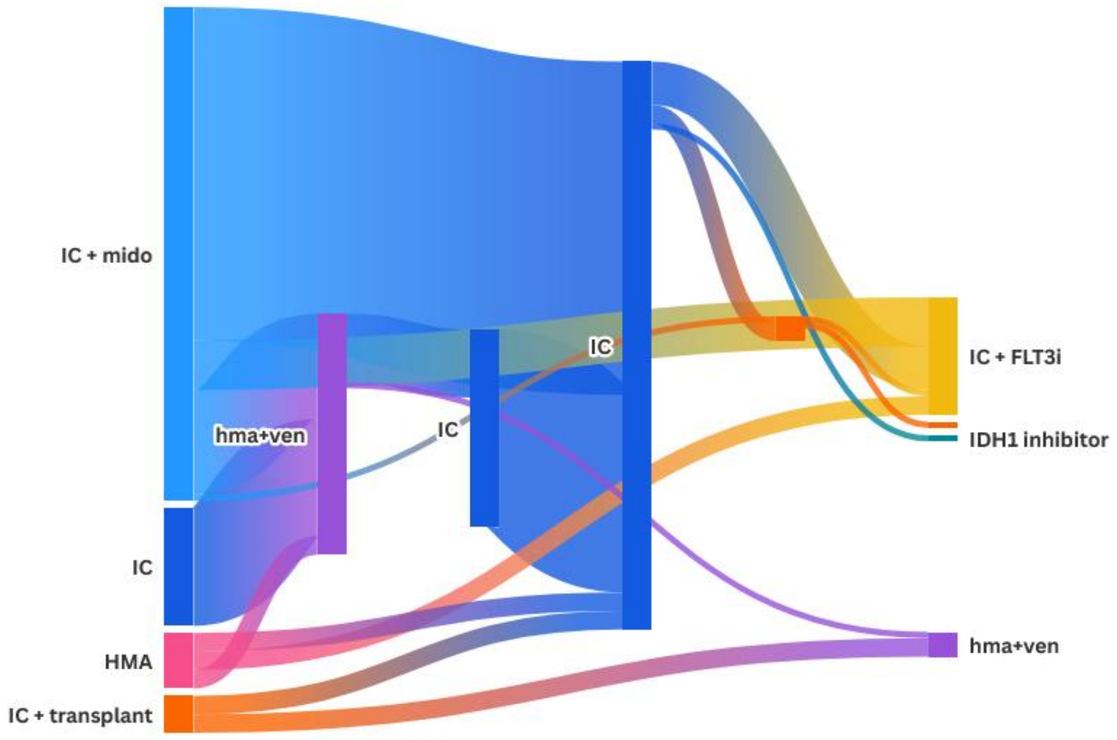

Supplement: Supplementary file 1 — Data S1: ajh70142‐sup‐0001‐Supinfo1.pdf. [file AJH-101-89-s001.pdf]
